# Effects of Ginseng Ingestion on Salivary Testosterone and DHEA Levels in Healthy Females: An Exploratory Study

**DOI:** 10.3390/nu12061582

**Published:** 2020-05-28

**Authors:** Emad A. S. Al-Dujaili, Maha N. Abu Hajleh, Ruth Chalmers

**Affiliations:** 1 Centre for Cardiovascular Science, Queen’s Medical Research Institute, University of Edinburgh, Edinburgh EH16 4TJ, UK; 2 Department of Pharmaceutical sciences, Faculty of Pharmacy, University of Jordan, Amman 11942, Jordan; mahaabuhajleh@hotmail.com; 3 Biological Sciences, Queen Margaret University, Edinburgh EH21 6UU, UK; rchalmers@hotmail.com

**Keywords:** ginseng, ginsenosides, adaptogen, testosterone, DHEA, androgens

## Abstract

Ginseng is a traditional herbal adaptogen that has been historically used in China and the Far East. Ginsenosides are the active component of ginseng known to exert several actions by targeting “multi-receptor systems”, both extracellular and intracellular. In humans, ginseng effects remain unclear. This study aimed to investigate whether ginseng can influence salivary androgen levels (testosterone and dehydroepiandrosterone (DHEA)) in females. The study followed a parallel partially controlled design. Healthy women (*n* = 24) were recruited and divided into two groups (A = 20–32 and B = 38–50 years). Volunteers were asked to maintain a food diary pre and post ginseng consumption and collected four salivary samples (7 a.m., 9 a.m., 12 p.m., and 5 p.m.) before and after ingesting 75 mg red Korean ginseng extract per day for seven days. Testosterone and DHEA were then assayed by ELISA methods. Group A’s mean daily salivary testosterone pre ginseng ingestion increased from 76.3 ± 16.6 to 98.4 ± 21.1 pg/mL post ginseng (*p* < 0.01) with significant difference at all time points, and mean daily salivary DHEA increased from 1.53 ± 0.63 to 1.98 ± 0.89 ng/mL post ginseng (*p* = 0.02). Group B’s mean daily salivary testosterone pre ginseng ingestion was 61.2 ± 16.9 and post ginseng 68.1 ± 11.5 pg/mL (*p* = 0.132), and daily salivary DHEA increased from 0.91 ± 0.32 to 1.62 ± 0.49 ng/mL post ginseng (*p* = 0.014) with significant difference at all time points. In conclusion, it appears that ginseng intake significantly increased salivary testosterone levels in the younger women group, but only slightly in the older group. However, DHEA levels in the older women showed a marked and significant increase. These results suggest a potential role for ginseng in modulating salivary androgen levels and that such effect may be more evident in older women where the levels of androgens (DHEA) start to decline. However, it has to be stressed that our results are preliminary and further properly controlled trials are justified.

## 1. Introduction

**Figure 1:**
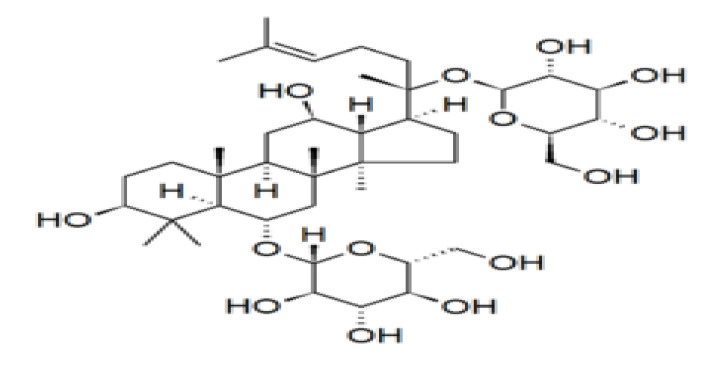
Chemical structure of the ginsenoside Rg1, a member of the dammarane family of molecules. Ginsenosides or panaxosides are a class of natural steroid glycosides and triterpene saponins found almost exclusively in the plant genus *Panax* (ginseng) [9].

Panax ginseng or Korean ginseng (Araliaceae) has been used as herbal medicine for more than 2000 years [1], and utilized as an adaptogenic agent to enhance resistance to stress and aging and improve physical performance [2,3,4]. Ginseng has numerous pharmacological effects on the reproductive, cardiovascular, endocrine, and immune systems. It has the ability to reduce fatigue, improve blood circulation, relieve menopausal symptoms, boost immune function, and influence steroid hormone levels [5,6,7]. Shibata et al. [8] isolated and identified the structures of ginseng saponins from the plant roots in 1963. Ginsenosides are steroidal saponins that have the four trans-ring rigid steroid skeleton with a variety of sugar moieties (see Figure 1) [4,9,10] and they represent the active constituents of panax ginseng. Ginsengs contain approximately 200 substances, such as ginsenosides, polysaccharides, polyacetylenes, peptides, amino acids, polyacetylenic alcohols, and fatty acids [1,4,8]. Currently, there are more than 26 isolated ginsenosides [11] that differ primarily in the number and position of sugar conjugates [9,10,11]. Ginsenoside Rh_2_, Rh_1_, F_1_, Rb_1_, and Rb_2_ and protopanaxatriol ginsenoside Rg_1_ are the most abundant types [1,4]. Ginsenosides activate the genomic pathway by binding the intracellular nuclear hormone receptors such as the glucocorticoid receptor, progesterone receptor, androgen receptor, mineralocorticoid receptor, estrogen receptor, peroxisome proliferator-activated receptor, and liver X receptor [12,13,14,15,16,17,18,19,20,21]. Ginsenosides and steroid hormones have similar structural characteristics; both are lipid-soluble signaling molecules that initiate a cellular response [8,9]. Ginsenoside Rb1 can bind and activate the estrogen receptor and other steroid receptors, including those of testosterone and other steroids [9]. Dehydroepiandrosterone (DHEA) and its sulphated ester, Dehydroepiandrosterone sulphate DHEAS are the precursors of testosterone produced primarily by the adrenal cortex, and are secretary products of the adrenal zona reticularis [22]. DHEA can be derived from circulating DHEAS catalyzed by steroid sulphatase [23]. DHEA and DHEAS are pro-androgens [22,24] and are precursors for other androgens and estrogens including testosterone and estradiol plus several other actions in the body. Other actions reported include the use of ginseng for the treatment of osteoporosis [25], inhibition of platelet aggregation [26], prevention of Alzheimer’s disease through the protection against beta-amyloid peptide-induced neuronal apoptosis [13,27], anti-oxidative stress effect of red ginseng in brain [18], inhibition of apoptosis in neuroblastoma cells and human breast cancer [28,29], and protection against testicular damage and gene expression in rats [30]. In women, 75% of premenopausal estrogens [31] and 100% of postmenopausal estrogens originate from DHEA [32]. It is now known that the levels of both DHEA and DHEAS in serum decline with age, and thus DHEA supplementation could improve ovarian function, increase pregnancy chances, and lower miscarriage rates in women [32,33]. 

Testosterone is produced in the testes of males and ovaries of females. However, testosterone can also be synthesized peripherally from DHEA by intracellular conversion. Transformation of DHEA and DHEAS depends on the expression of various steroidogenic enzymes [24]. Salivary testosterone represents the concentration of bio-available testosterone. Even though DHEAS exceeds the concentration of DHEA by approximately 300–500 times [34], the salivary DHEA represents bioavailable DHEA and not DHEAS. The unconjugated DHEA enters saliva by intracellular diffusion and represents the concentration of unbound active DHEA in plasma [35]. The aim of this study was to determine if ginseng intake could influence salivary testosterone and DHEA in healthy females. The ginsenoside content varies considerably in over-the-counter products. This study used Korean ginseng prepared from the dried roots of the species *Panax* C.A. Meyer (Red Kooga Korean Ginseng) which contains not less than 10% of ginsenosides.

## 2. Materials and Methods

### 2.1. Materials

Korean red ginseng manufactured by Red Kooga was purchased from Superdrug, Edinburgh, UK. Each capsule contained 75 mg of ginseng extract (equivalent to 600 mg ginseng root powder) with a guaranteed ginsenoside content of 7.5 mg. Diethyl ether and methanol were obtained from Fisher Scientific, Loughborough, UK. Sheep anti-DHEA antibody (used at a final dilution of 1:40,000), anti-testosterone antibody (used at a final dilution of 1:200,000), and horseradish peroxidase-donkey sheep anti-sheep conjugate (used at a concentration of 1: 10,000) were purchased from Micropharm Ltd., Newcastle Emlyn, UK. Testosterone and DHEA standards, Tween 20, sulfuric acid, tetra-methyl-benzidine, bovine serum albumin were purchased from Sigma-Aldrich, Poole, UK. ELISA plates were obtained from Griener Bio-One, Frickenhausen, Germany. 

### 2.2. Study Design

The study followed a modified parallel and partially controlled placebo design where the participants chose to take the ginseng capsules and some then volunteered to take the placebo (maltodextrin) similar to the ginseng capsules. Information sheets were provided to all potential volunteers and written informed consent from each participant was obtained prior to participation. The study was granted ethical approval by the Divisional Ethics Committee at Queen Margaret University, Edinburgh, United Kingdom, code: 02022630/2012-HONORS/ GINSENG/DNBS/QMU Ethical Committee. The intervention was conducted according to the guidelines laid down in the Declaration of Helsinki [36]. All collected data were stored according to the Data Protection Act (1998) [37].

### 2.3. Subject Recruitment

Participants were recruited from the local community (including students and staff) through advertising in the Queen Margaret University research recruitment digest and by word-of-mouth. Eligible participants included women, aged 20–50 years with a BMI between 18 and 34.9 kg/m². Volunteers answered the pre-assessment questionnaire before they registered for the study to ensure they did not have any symptomatic disease. Exclusion criteria included taking medication for diabetes, heart, liver, or kidney disease. Pregnant and lactating women and those with allergies to ginseng were also excluded. Subjects (*n* = 24; 12 were Queen Margaret students and 12 were Queen Margaret staff) were stratified according to age into 2 groups: Group A was composed of 12 subjects aged 20–32 years and Group B was composed of another 12 subjects aged 38–50 years. 

### 2.4. Ginseng Supplement

Red Kooga Korean red ginseng was used and standardized so that each capsule contained 75 mg ginseng extract (equivalent to 600 mg ginseng root powder) with a guaranteed ginsenoside content of 7.5 mg [6]. Chemical analysis of a ginseng capsule was not preformed, thus the ginsenoside content was according to the manufacturer’s label. It is known that Korean ginseng generally consists of the main root and the lateral or fine roots at a ratio of about 3:1. The *Panax* red ginseng used in our study was produced according to the method previously developed by Lee et al. [38]. The consort statement for the reporting of herbal trials was followed [39]. The ginsenoside content of the red ginseng used (mg/g) was as follows: Rg1 = 3.3, Re = 2.0, Rb1 = 5.8, Rc =1.7, Rb2 = 2.3, and Rd = 0.4. Each subject consumed 1 ginseng capsule orally for seven days. However, Red Kooga recommends 1 capsule should be consumed orally for eight weeks. 

### 2.5. Study Protocol

Subjects collected basal saliva samples (3 mL) at 7 a.m., 9 a.m., 12 p.m., and 5 p.m. before the intake of ginseng and kept a food diary for 48 hours (including 1 weekday and 1 weekend day). Subjects then consumed one 75 mg ginseng capsule extract daily for 7 days and maintained a second 48-hour food diary (including 1 weekday and 1 weekend day). Saliva samples (3 mL) were also collected on the final day of ginseng supplementation at 7 a.m., 9 a.m., 12 p.m., and 5 p.m. All subjects decided to take ginseng capsules (one capsule per day) for one week, followed by the collection of saliva samples and the diet diaries. All subjects were first supplied with 7 ginseng capsules, 1 packet sugar free chewing gum, 8 labelled collection tubes and an information sheet on saliva sample collection. For the placebo arm, 6 subjects from each group then volunteered to take a placebo of maltodextrin similar to the ginseng capsules following a washout period of 1 week. They were then supplied with the same things as before.

### 2.6. Diet Diaries

To account for any changes in energy and macronutrient intake (carbohydrate, protein, fat, and total energy) that might influence the results, a two-day food diary was collected at baseline and over the same days of the intervention week. Subjects completed a four-day diet diary, that is, 48 hours pre ginseng (including 1 weekday and 1 weekend day) and similarly 48 hours post ginseng. Nutrient intakes were generated using the Win Diets Research software program (Version 2010, Robert Gordon University, Aberdeen, UK). Food recording support and training, including guidance on portion sizes and household measures, were provided by the researcher to assist participants in completing the food diaries. 

### 2.7. Sample Preparation and Hormone Estimation

Saliva samples were frozen immediately following collection at –20 °C. Then, they were thawed and centrifuged at 3600 rpm for 10 minutes to remove debris, and 3 aliquots of 700 µL in Eppendorf tubes were labelled and frozen at –20 °C until required. Samples containing pre and post ginseng saliva were defrosted and centrifuged at 2600 *g* for 3 minutes, and 0.5 mL of each sample was extracted with diethyl ether for 10 minutes [23]. The ether layer was then evaporated under nitrogen at 45 °C and the residue was reconstituted in 0.5 mL of assay buffer (PBS + 0.1% BSA). Testosterone and DHEA were then assayed by highly specific and sensitive ELISA standard protocols previously described by Al-Dujaili and colleagues [23,40]. 

### 2.8. Statistical Analysis

Data were analyzed using Microsoft Excel and SPSS for Windows version 21.0 (SPSS, Chicago, IL, USA) and expressed as mean ± SD or SEM. Differences in baseline characteristics were examined using independent *t*-tests. For multiple comparisons, data were analyzed using two-way mixed model analysis of variance (ANOVA) with time (baseline, week 1 post ginseng) as within-subject factor, and treatment (ginseng/placebo) as between-subject factor. Energy, protein, fat, and carbohydrate intakes were analyzed using paired *t*-tests. Significance was set at *p* ≤ 0.05. 

## 3. Results

All volunteers recruited completed the study. Mean age (± SD) of group A was 23.2 ± 3.6 years and of group B was 42.8 ± 4.3 years (*p* < 0.001). Weight and height were estimated enabling a body mass index (BMI) calculation. Group A recorded a BMI value of 23.6 ± 3.2, and group B’s BMI was 26.9 ± 4.4 (mean ± SD). An independent paired *t*-test was preformed determining no significant difference between the weight (*p* = 0.276) or BMI (*p* = 0.075) between groups A and B. This was a desirable result as the authors contend that the androgen levels obtained were not significantly influenced by weight. None of our female subjects were taking oral contraceptive (OC) medications. This was important as researchers have reported that OC medications decrease bio-available testosterone, DHEA, and DHEAS [41,42]. Alcohol intake is known to affect steroid hormone levels. However, subjects agreed not to consume excessive amounts of alcohol during the study and alcohol consumption was prohibited 24 hours prior to saliva collection. Therefore, it was assumed that alcohol did not influence the results of this study.

Salivary testosterone and DHEA decrease with age as reported by many authors [43,44]. The mean daily salivary testosterone pre (76.3 pg/mL) and post (98.4 pg/mL) ginseng supplementation (*p* < 0.01) in individuals aged from 20 to 32 years was greater than the mean daily salivary testosterone pre (61.6 pg/mL) and post (68.40 pg/mL) ginseng supplementation (*p* = 0.132) in individuals aged from 38 to 50 years. Similarly, the production of DHEA and DHEAS was found to be reduced with age as previously reported [24,45]. The mean daily salivary DHEA pre (1.53 ng/mL) and post (1.98 ng/mL) ginseng supplementation (*p* = 0.02) in individuals between 20 and 32 years was greater than the mean daily salivary DHEA pre (0.91 ng/mL) and post (1.62 ng/ml) ginseng supplementation (*p* = 0.014) in individuals aged between 38 and 50 years (see Table 1). As shown in Table 1, there was a marked increase in the mean daily salivary testosterone in group A subjects (*p* < 0.01) post ginseng intake. However, in group B subjects, there was a slight increase in mean daily salivary testosterone but it was not significant (*p* = 0.132). In contrast, mean daily salivary DHEA increased significantly following ginseng intake in group A (*p* = 0.02) and group B (*p* = 0.014) volunteers. 

**Figure 2:**
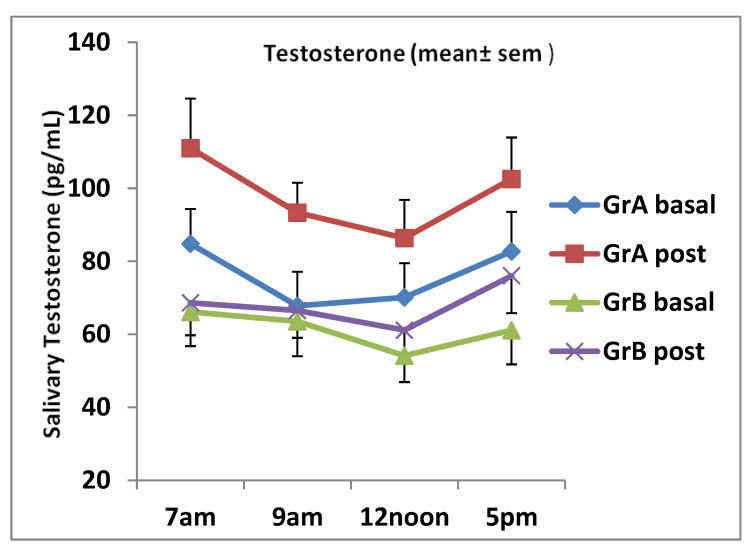
Daily salivary testosterone levels (mean ± SEM) for individuals aged 20–32 years (group A) and those aged 32–50 years (group B) pre and post ginseng supplementation. There were significant increases in testosterone at all time points in group A: 7 a.m., *p* < 0.01; 9 a.m., *p* = 0.0031; 12 noon, *p* = 0.026, 5 p.m., *p* = 0.044. There was no significant increase at all times in group B.

**Figure 3:**
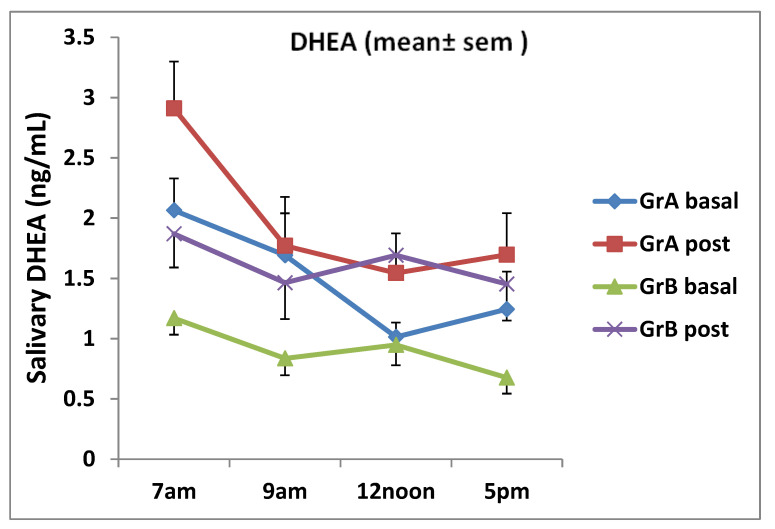
Daily salivary dehydroepiandrosterone (DHEA) testosterone levels for individuals aged 20–32 years (group A) and those aged 38–50 years (group B) pre and post ginseng intake (mean ± SEM). There was a significant increase in DHEA at 7 a.m. only in group A (*p* = 0.017). It was determined that mean DHEA in group B increased at all time points (7 a.m., *p* = 0.05; 9 a.m., *p* = 0.037; 12 noon, *p* = 0.04, 5 p.m., *p* = 0.046).

Steroid hormones demonstrate circadian rhythm as confirmed by several studies [43,45,46]. Mean salivary testosterone and DHEA levels at 7 a.m. were the highest in our study for individuals aged between 20 and 30 years and those aged between 38 and 50 years at baseline (Figure 2 and Figure 3). The levels for both steroids post ginseng supplementation reduced up to 12 noon, and then they tended to fluctuate following that time. At 5 p.m., salivary testosterone showed an increase as well as the DHEA levels in group A subjects. However, in group B subjects, the DHEA levels continued to decrease (see Figure 2 and Figure 3). It seems that the erogenic effect of ginseng on DHEA in women aged between 38 and 50 years was greater than that in women aged between 20 and 32 years, although no significant difference was observed. Salivary testosterone and DHEA samples obtained from subjects who performed the placebo were measured by the same ELISA methods. The placebo data were very similar to those obtained by groups A and B volunteers at basal and were not statistically different from those obtained pre ginseng consumption. Therefore, placebo data comparisons were performed with post ginseng values using ANOVA and they revealed that the results obtained were not statistically different when pre and post ginseng data were analyzed. 

Analysis of food intake was performed using SPSS to determine if there was a significant difference between energy, carbohydrate, fat, and protein consumed pre and post ginseng in groups A and B. There were no significant differences in group A’s macronutrient intake between pre and post ginseng supplementation (Table 2). However, the energy (*p* = 0.05) and fat (*p* = 0.041) intakes of group B were significantly different pre and post ginseng supplementation, presumably due to an increase in fat intake (Table 2). 

## 4. Discussion

There has been a paucity of studies investigating the effect of ginseng consumption on female testosterone and DHEA serum and saliva levels. To our knowledge, the results of this study seem to be novel. Nevertheless, the results support available scientific literature, suggesting that salivary testosterone and DHEA levels decrease with age as reported by the authors of [24,44]. Labrie et al. [24] also reported that the formation of DHEA and DHEAS declines with age, as do other researchers [41,43]. Furthermore, our results indicate that an ergogenic effect of ginseng intake on salivary DHEA occurred in women, and that it was greater in women aged 38–50 years than those aged 20–32 years. 

Subjects were responsible for daily ginseng consumption; consequently, the authors rely on the assumption that each subject complied. Furthermore, a review of scientific evidence has highlighted that the absorption and bioavailability of ginsenosides in humans are not yet clear [6,47].

Our study is a preliminary and exploratory trial, and we searched the literature and found that a 600 mg dose of ginseng root powder used by several studies and universally accepted does not produce side effects. We used 600 mg of Red Kooga Korean ginseng dried powder, which contains 75 mg of standardized ginseng extract with a guaranteed ginsenoside content of 10%. Future trials will investigate the optimal dose condition of the dose–response relationship. One of the limitations of our study was that randomization was not properly implemented. Randomization was employed within each age group. However, following the completion of the study, we realized that only six volunteers took the placebo. Nevertheless, we completed the analysis and we did not observe any significance in all outcomes between the basal data of those taking the ginseng in both age groups and the data of post placebo intervention. This study identified the need for an adequately powered clinical trial to substantiate our results and establish the degradation pathways of ginsenosides in humans. Nevertheless, the results suggest that ginseng intake influences salivary androgenic hormones in female individuals. Saliva was the specimen collected as it was obtained by a non-invasive technique [48]. Moreover, multiple specimens could be obtained from the same individual allowing hormonal circadian rhythm identification; serum androgen concentration determination would have been invasive and impractical. In addition, several researchers have reported that saliva is a reliable diagnostic specimen [43,45,48,49,50].

Salivary testosterone represents the concentration of bio-available testosterone. However, salivary DHEA represents bio-available DHEA and not DHEAS [51,52]. Levels of DHEA-S in saliva are compromised by the method of entry (ultrafiltration), as DHEA-S is a conjugated hormone. Unconjugated DHEA enters saliva simply by intracellular diffusion and represents the concentration of the unbound active DHEA in plasma [48,53]. Testosterone circulates in women plasma bound strongly to sex hormone binding globulin (SHBG) (66–74%) and 24–30% bound weakly to albumin with only about 1–3% being free in the blood [54,55]. Under certain conditions the bio-available testosterone dissociates from its carrier protein (mostly albumin) and becomes free.

Steroid hormones in the periphery are believed to equilibrate rapidly between tissues and blood [56,57]. Total concentrations of testosterone in peripheral tissue and body fluids are mainly dependent upon the levels of binding proteins such as SHBG and albumin. These binding proteins can act as a reservoir for the steroid and protect it against extensive metabolism of active (free) steroids during passage of the blood through the liver [58]. Recently, salivary and serum testosterone levels were found to correlate significantly with calculated free testosterone in both controls and patients, whereas salivary and urine testosterone showed weaker correlations [59]. Other researchers reported that the correlation of serum and salivary concentrations taken before and after exercise was not as consistent as that observed during rest [60]. However, Lane and Hackney [61] reported a strong correlation observed between salivary and serum testosterone concentrations when exercising at both moderate and high intensities.

Diet diaries were used to show that there was no significant difference between nutrient intake pre and post ginseng intervention when using a weighed intake or a diet diary [62]. It has been reported that carbohydrate, fat, and protein intake can influence androgen production [63,64]. Group A subjects’ intakes of energy, carbohydrate, fat, and protein were not significantly different pre and post ginseng supplementation. However, post ginseng supplementation energy and fat intake was slightly increased in group B subjects. These were analyzed using Windiet software that may have produced some errors as some foods consumed were often not listed and therefore substitute foods were used. 

The ELISA methods used to determine the levels of testosterone and DHEA were carefully developed and optimized by our in-house immunoassay laboratory [23,40,50]. These highly specific and sensitive assays are fully validated methods for the determination of salivary testosterone and DHEA. DHEAS, a conjugated hydrophilic steroid, enters saliva via ultrafiltration unlike DHEA, an unconjugated hydrophobic steroid, which enters saliva by intracellular diffusion [35]. The hydrophilic properties of DHEAS result in DHEAS being discarded in the aqueous solution during diethyl ether extraction [23]. Therefore, the concentration of DHEA in saliva truly represents the concentration of bio-available DHEA without DHEAS. Salivary analysis of DHEA has shown a significant positive correlation with plasma DHEA levels [65,66]. Salivary detection of DHEAS, on the other hand, has been shown to have limited useful indications of plasma DHEA levels due to low concentrations available in saliva, which can be readily affected by trace contamination [35]. Normal salivary testosterone and DHEA levels differ according to the individual laboratory. Our overall normal ranges for female salivary testosterone (18–69 years) were 58–246 pg/mL (a.m.) and 32–166 pg/mL (p.m.), and those for DHEA were 115–674 pg/mL (a.m.) and 62–390 pg/mL (p.m.) [45,50]. A review of available literature highlights that contraceptive medication decreases bio-available testosterone and DHEA [42]. However, none of our volunteers were taking oral contraceptives. Similarly, subjects agreed not to consume excessive amounts of alcohol during the study and alcohol consumption 24 hours prior to saliva collection was prohibited. Therefore, it was assumed that alcohol did not influence the results of the study. 

Steroid hormones demonstrate daily circadian rhythm as reported by many researchers [43,45,46]. These finding are supported by the fact that the secretion of androgens produced by the zona reticularis follows the actions of the adrocorticotrophic hormone (ACTH) on the adrenal gland. Mean salivary testosterone and DHEA concentrations in subjects aged 20–32 years pre and post ginseng intake showed a daily circadian rhythm as well as in subjects aged 38–50 years, although at a reduced pattern (see Figure 1 and Figure 2). Our study showed that mean salivary testosterone levels started to increase at 5 p.m. pre and post ginseng intake. This has been previously observed in some women due to increased physical activity, particularly in younger women [22,67,68], or presumably due to the decreased metabolism of testosterone caused by ginseng in older women and the low number of subjects in the study. However, salivary DHEA showed a typical daily circadian rhythm, which follows the ACTH secretion [22,31]. The basal mean ± SD salivary testosterone concentration for women aged 20–32 years at 7 a.m. was 84.8 ± 33.3pg/mL and, for women aged 32–50 years, it was 66.4 ± 27.6 pg/mL, which were expected and acceptable values. Furthermore, the mean ± SD basal DHEA levels at 7 a.m. were 2.06 ± 0.92 (group A) and 1.17 ± 0.45 (group B) [22,45,50].

Following ginseng intake, there was a marked increase in the mean daily salivary testosterone in group A. However, in group B subjects, there was a slight increase in mean daily salivary testosterone. In contrast, the mean daily salivary DHEA increased significantly following ginseng intake in groups A and B volunteers. To our knowledge, these results appear to be novel and it is worth conducting future studies using increased participation to determine if ginseng significantly increases testosterone and DHEA concentrations in women. If a ginseng supplement has the potential to increase salivary testosterone and DHEA, in particular, it could be beneficial to postmenopausal women and those with suspected insufficiency, as suggested by several researchers who reported that DHEA is beneficial when administered during menopause [24,33,69] as a substitute for androgen replacement therapy. Proposed mechanisms of action of ginsenosides could be due to several hypotheses, including an inhibitory activity of the enzymes that glucoronidate testosterone during metabolism and excretion in a way similar to the action of other compounds [70]. Moreover, the increase in DHEA levels could be attributed to the inhibition of the SULT2A1 enzyme that is responsible for the sulfonation of DHEA into DHEAS [23]. Furthermore, we can hypothesize that the erogenic effect of ginseng in women aged 38–50 years was greater possibly due to the delayed metabolism of ginsenosides in this group. However, the same cannot be said for testosterone.

## 5. Conclusions

It appears that a one-week ingestion of Korean ginseng slightly but significantly increased salivary testosterone and DHEA in younger women (aged 20–32 years) and salivary DHEA in older women (aged 38–50 years). However, it must be stressed that our results are preliminary and further properly controlled trials are justified. These data suggest a role for ginseng to play in modulating salivary androgen levels and that such an effect may be more evident in women above the age of 40 years where the levels of androgens, and particularly DHEA, start to decline. Such an erogenic effect could be beneficial to women when ginseng is administered to postmenopausal women in low doses. Future investigations are required to verify the type of ginsenosides that produces the erogenic effect in humans, identify the mechanism of action by which ginseng exerts its health benefits, and investigate gender, age, and BMI parameters. 

## Figures and Tables

**Table 1:** Average daily salivary steroid values of groups A and B volunteers pre and post ginseng consumption.

Average Daily Value	Pre Ginseng	± SD	Post Ginseng	± SD	*p*-value
Group A Testo (pg/mL)	76.3	16.6	98.4	21.1	< 0.01
Group B Testo (pg/mL)	61.6	16.9	68.4	11.5	0.132
Group A DHEA (ng/mL)	1.53	0.63	1.98	0.89	0.02
Group B DHEA (ng/mL)	0.91	0.32	1.62	0.49	0.014

**Table 2:** Comparison of macronutrient intake (energy, protein, carbohydrate, and fat) of groups A and B volunteers pre and post ginseng supplementation.

Nutrient		Mean	± SD	Paired *t*-test
Group A				
Energy (kcal)	Pre	1610.46	336.701	0.376
	Post	1446.11	384.541	
Protein (g)	Pre	64.44	16.704	0.435
	Post	58.22	23.419	
Carbohydrate (g)	Pre	216.11	74.824	0.323
	Post	187.00	36.166	
Fat (g)	Pre	59.44	18.290	0.500
	Post	54.44	24.501	
Group B				
Energy (kcal)	Pre	1696.25	258.578	0.050
	Post	2030.38	382.317	
Protein (g)	Pre	72.25	16.968	0.147
	Post	83.38	17.541	
Carbohydrate (g)	Pre	229.00	42.007	0.281
	Post	247.88	38.698	
Fat (g)	Pre	56.58	17.883	0.041
	Post	81.25	33.856	

## Abbreviations

ACTH	Adrocorticotrophic hormone
BMI	Body mass index
DHEA	Dehydroepiandrosterone
DHEAS	Dehydroepiandrosterone sulphate
ELISA	Enzyme-linked immunosorbent assay
SHBG	Sex hormone binding globulin
SPSS	Statistical Package for the Social Sciences
Testo	Testosterone

## References

[1] Li C.P., Li R.C. (1973). An Introductory Note to Ginseng. Am. J. Chin. Med..

[2] Singh V.K., Agarwal S.S., Gupta B.M. (1984). Immunomodulatory activity of Panax ginseng extract. Planta Medica.

[3] Scaglione F., Ferrara F., Dugnani S., Falchi M., Santoro G., Fraschini F. (1990). Immunomodulatory effects of two extracts of Panax ginseng CA Meyer. Drugs Exp. Clin. Res..

[4] Kim D.H. (2012). Chemical diversity of Panax ginseng, Panax quinquifolium, and Panax notoginseng. J. Ginseng Res..

[5] Park H.J., Kim N.H., Park S.J., Kim J.M., Ryu J.H. (2012). Ginseng in traditional herbal prescriptions. J. Ginseng Res..

[6] Park J., Song H., Kim S.-K., Lee M.S., Rhee D.K., Lee Y. (2016). Effects of ginseng on two main sex steroid hormone receptors: Estrogen and androgen receptors. J. Ginseng Res..

[7] Kang S., Min H. (2012). Ginseng, the ‘immunity boost’: The effects of Panax ginseng on immune system. J. Ginseng Res..

[8] Shibata S., Tanaka O., Soma K., Aando T., Iida Y., Nakamura H. (1965). Studies on saponins and sapogenins of ginseng. The structure of panaxatriol. Tetrahedron Lett..

[9] Attele A.S., Wu J.A., Yuan C.S. (1999). Ginseng pharmacology: Multiple constituents and multiple actions. Biochem. Pharmacol..

[10] Harkey M.R., Henderson G.L., Gershwin M.E., Stern J.S., Hackman R.M. (2001). Variability in commercial ginseng products: An analysis of 25 preparations. Am. J. Clin. Nutr..

[11] Park C.S., Yoo M.H., Noh K.-H., Oh D.K. (2010). Biotransformation of ginsenosides by hydrolyzing the sugar moieties of ginsenosides using microbial glycosidases. Appl. Microbiol. Biotechnol..

[12] Chung E., Lee K.Y., Lee Y., Lee Y.H., Lee S.K. (1998). Ginsenoside-Rg1 down-regulates glucocorticoid receptor and displays synergistic effects with cAMP. Steroids.

[13] Wu J., Pan Z., Wang Z., Zhu W., Shen Y., Cui R., Lin J., Yu H., Wang Q., Yu Y. (2012). Ginsenoside Rg1 protection against β-amyloid peptide-induced neuronal apoptosis via estrogen receptor α and glucocorticoid receptor-dependent anti-protein nitration pathway. Neuropharmacology.

[14] Shen K., Leung S.W., Ji L., Huang Y., Hou M., Xu A., Wang Z., Vanhoutte P.M. (2014). Notoginsenoside Ft1 activates both glucocorticoid and estrogen receptors to induce endothelium-dependent, nitric oxide-mediated relaxations in rat mesenteric arteries. Biochem. Pharmacol..

[15] Bae J.-S., Park H.S., Park J.W., Li S.H., Chun Y.-S. (2011). Red ginseng and 20 (S)-Rg3 control testosterone-induced prostate hyperplasia by deregulating androgen receptor signaling. J. Nat. Med..

[16] Wang W., Wang H., Rayburn E.R., Zhao Y., Hill D.L., Zhang R. (2008). 20-(S)-25-methoxyl-dammarane-3β, 12β, 20-triol, a novel natural product for prostate cancer therapy: Activity in vitro and in vivo and mechanisms of action. Br. J. Cancer.

[17] Park G.H., Park K.Y., Cho H.-I., Lee S.M., Han J.S., Won C.H., Shin H. (2015). Red ginseng extract promotes the hair growth in cultured human hair follicles. J. Med. Food.

[18] Kim E.H., Kim I.H., Lee M.J., Nguyen C.T., Ha J.A., Lee S.C., Choi S., Choi K.T., Pyo S., Rhee D.K. (2013). Anti-oxidative stress effect of red ginseng in the brain is mediated by peptidyl arginine deiminase type IV (PADI4) repression via estrogen receptor (ER) β up-regulation. J. Ethnopharmacol..

[19] Ding J., Xu Y., Ma P., Jinna A., Yang X., Liu Z., Lin N. (2015). Estrogenic effect of the extract of Renshen (Radix Ginseng) on reproductive tissues in immature mice. J. Tradit. Chin. Med..

[20] Xu Y., Ding J., Ma X.-P., Ma Y.-H., Liu Z.Q., Lin N. (2014). Treatment with Panax ginseng antagonizes the estrogen decline in ovariectomized mice. Int. J. Mol. Sci..

[21] Shim M.K., Lee Y. (2012). Estrogen Receptor Is Activated by Korean Red Ginseng In Vitro but Not In Vivo. J. Ginseng Res..

[22] Burger H.G. (2002). Androgen production in women. Fertil. Steril..

[23] Al-Dujaili E.A.S., Kenyon C.J., Nicol M.R., Mason J.I. (2011). Liquorice and glycyrrhetinic acid increase DHEA and deoxycorticosterone levels in vivo and in vitro by inhibiting adrenal SULT2A1 activity. Mol. Cell. Endocrinol..

[24] Labrie F., Luu-The V., Lin S.-X., Simard J., Pelletier G., Labrie C. (2005). Is dehydroepiandrosterone a hormone?. J. Endocrinol..

[25] Siddiqi M.H., Siddiqi M.Z., Ahn S., Kang S., Kim Y.J., Sathishkumar N., Yang N.U., Yang D.C. (2013). Ginseng saponins and the treatment of osteoporosis: Mini literature review. J. Ginseng Res..

[26] Lee D.H., Cho H.J., Kim H.H., Rhee M.H., Ryu J.H., Park H.J. (2013). Inhibitory effects of total saponin from Korean red ginseng via vasodilator-stimulated phosphoprotein-Ser157 phosphorylation on thrombin-induced platelet aggregation. J. Ginseng Res..

[27] Zhang X., Wang J., Xing Y., Gong L., Li H., Wu Z., Li Y., Wang J., Wang Y., Dong L. (2012). Effects of ginsenoside Rg1 or 17beta-estradiol on a cognitively impaired, ovariectomized rat model of Alzheimer’s disease. Neuroscience.

[28] Nguyen C.T., Luong T.T., Kim G.L., Pyo S., Rhee D.K. (2014). Korean Red Ginseng inhibits apoptosis in neuroblastoma cells via estrogen receptor β-mediated phosphatidylinositol-3 kinase/Akt signaling. J. Ginseng Res..

[29] Gao Q.G., Chan H.Y., Man C.W.-Y., Wong M.S. (2014). Differential ERα-mediated rapid estrogenic actions of ginsenoside Rg1 and estren in human breast cancer MCF-7 cells. J. Steroid Biochem. Mol. Boil..

[30] Kopalli S.R., Won Y.J., Hwang S.Y., Cha K.M., Kim S.Y., Han C.K., Lee S.H., Hong J.Y., Kim S.-K. (2016). Korean red ginseng protects against doxorubicin-induced testicular damage: An experimental study in rats. J. Funct. Foods.

[31] Payne A.H., Hales D.B. (2004). Overview of steroidogenic enzymes in the pathway from cholesterol to active steroid hormones. Endocr. Rev..

[32] Zhou Y., Kang J., Chen D., Han N., Ma H. (2015). Ample evidence: Dehydroepiandrosterone (DHEA) conversion into activated steroid hormones occurs in adrenal and ovary in female rat. PLoS ONE.

[33] Gleicher N., Barad D.H. (2011). Dehydroepiandrosterone (DHEA) supplementation in diminished ovarian reserve (DOR). Reprod. Boil. Endocrinol..

[34] Orentreich N., Brind J.L., Rizer R.L., Vogelman J.H. (1984). Age changes and sex differences in serum dehydroepiandrosterone sulfate concentrations throughout adulthood. J. Clin. Endocrinol. Metab..

[35] Vining R.F., McGinley R.A., Symons R.G. (1983). Hormones in saliva: Mode of entry and consequent implications for clinical interpretation. Clin. Chem..

[36] Association W.M., World Medical Association Declaration of Helsinki (2001). Ethical principles for medical research involving human subjects. Bull. World Health Organ..

[37] Legislation (1998). Data Protection Act. http://www.legislation.gov.uk/ukpga/1998/29/contents.

[38] Lee S.M., Bae B.-S., Park H.W., Ahn N.G., Cho B.G., Cho Y.L., Kwak Y.S. (2015). Characterization of Korean Red Ginseng (*Panax ginseng* Meyer): History, preparation method, and chemical composition. J. Ginseng Res..

[39] Altman D.G., Schulz K.F., Moher D., Egger M., Davidoff F., Elbourne D., Gøtzsche P.C., Lang T. (2001). The revised consort statement for reporting randomized trials: Explanation and elaboration. Ann. Intern. Med..

[40] Al-Dujaili E.A.S. (2006). Development and validation of a Simple and Direct ELISA method for the determination of conjugated (glucuronide) and non-conjugated Testosterone excretion in urine. Clin. Chim. Acta.

[41] Bancroft J., Sherwin B.B., Alexander G., Davidson D.W., Walker A. (1991). Oral contraceptives, androgens, and the sexuality of young women: II. The role of androgens. Arch. Sex. Behav..

[42] Coenen C.M., Thomas C.M., Borm G.F., Hollanders J.M., Rolland R. (1996). Changes in androgens during treatment with four low-dose contraceptives. Contraception.

[43] Al-Dujaili E.A.S., Sharp M. Establishing Circadian Rhythm Profiles for Salivary Testosterone in Women: Evidence of Decline during Aging. Proceedings of the 8th European Congress of Endocrinology.

[44] Zumoff B., Strain G.W., Miller L.K., Rosner W. (1995). Twenty-four-hour mean plasma testosterone concentration declines with age in normal premenopausal women. J. Clin. Endocrinol. Metab..

[45] Al-Turk W., Al-Dujaili E.A.S. (2016). Effect of age, gender and exercise on salivary dehydroepiandrosterone circadian rhythm profile in human volunteers. Steroids.

[46] Kiss Z., Ghosh P.M. (2016). Women in cancer thematic review: Circadian rhythmicity and the influence of ‘clock’ genes on prostate cancer. Endocr. Relat. Cancer.

[47] Tawab M.A., Bahr U., Karas M., Wurglics M., Schubert-Zsilavecz M. (2003). Degradation of ginsenosides in humans after oral administration. Drug Metab. Dispos..

[48] Granger D.A., Schwartz E.B., Booth A., Curran M., Zakaria D. (1999). Assessing dehydroepiandrosterone in saliva: A simple radioimmunoassay for use in studies of children, adolescents and adults. Psychoneuroendocrinology.

[49] Hofman L.F. (2001). Human saliva as a diagnostic specimen. J. Nutr..

[50] Al-Dujaili E.A.S., Sharp M.A., Ostojic S.M. (2012). Female Salivary Testosterone: Measurement, Challenges and Applications, Steroids—Physiology to Clinical Medicine.

[51] Vittek J., L’Hommedieu D.G., Gordon G.G., Rappaport S.C., Southren A.L. (1985). Direct radioimmunoassay of salivary testosterone: Correlation with free and total serum testosterone. Life Sci..

[52] Longcope C., Hui S.L., Johnston C.C. (1987). Free estradiol, free testostereone, and sex hormone-binding globulin in perimenopausal women. J. Clin. Endocrinol. Metab..

[53] Voegtline K.M., Granger D.A. (2014). Dispatches from the interface of salivary bioscience and neonatal research. Front. Endocrinol..

[54] Pardridge W.M., Demers L.M. (1991). Bioavailable testosterone in salivary glands. Clin. Chem..

[55] Vermeulen A., Verdonck L., Kaufman J.M. (1999). A critical evaluation of simple methods for the estimation of free testosterone in serum. J. Clin. Endocrinol. Metab..

[56] Goncharov N.P., Katsya G.V., Chagina N.A., Gooren L.J. (2009). Testosterone and obesity in men under the age of 40 years. Andrologia.

[57] Miller K.K., Rosner W., Lee H., Hier J., Sesmilo G., Schoenfeld D., Neubauer G., Klibanski A. (2004). Measurement of free testosterone in normal women and women with androgen deficiency: Comparison of methods. J. Clin. Endocrinol. Metab..

[58] Mendel C.M. (1989). The free hormone hypothesis: A physiologically based mathematical model. Endocr. Rev..

[59] Lood Y., Aardal-Eriksson E., Webe C., Ahlner J., Ekman B., Wahlberg J. (2017). Relationship between testosterone in serum, saliva and urine during treatment with intramuscular testosterone undecanoate in gender dysphoria and male hypogonadism. Andrology.

[60] Cadore E.L., Lhullier M., Brentano E., Silva M., Ambrosini M., Spinelli R., Silva R., Kruel L. (2008). Correlations between serum and salivary hormonal concentrations in response to resistance exercise. Sports Sci..

[61] Lane A.R., Hackney A.C. (2014). Relationship between salivary and serum testosterone levels in response to different exercise intensities. Hormones.

[62] Black A.E., Welch A., Bingham S.A. (2000). Validation of dietary intakes measured by diet history against 24 h urinary nitrogen excretion and energy expenditure measured by the doubly-labelled water method in middle-aged women. Br. J. Nutr..

[63] Al-Dujaili E.A.S., Bryant M.L. Effect of Meal Fat Content on Salivary Testosterone and Cortisol Levels in Healthy Female Volunteers. Proceedings of the 196th Meeting of the Society for Endocrinology and Society for Endocrinology joint Endocrinology and Diabetes Day.

[64] Berrino F., Bellati C., Secreto G., Camerini E., Pala V., Panico S., Allegro G., Kaaks R. (2001). Reducing bioavailable sex hormones through a comprehensive change in diet: The diet and androgens (DIANA) randomized trial. Cancer Epidemiol. Biomark. Prev..

[65] Gallagher P., Leitch M., Massey A.E., McAllister-Williams R.H., Young A.H. (2006). Assessing cortisol and dehydroepiandrosterone (DHEA) in saliva: Effects of collection method. J. Psychopharmacol..

[66] Ahn R.S., Lee Y.J., Choi J.Y., Kwon H.B., Chun S.I. (2007). Salivary Cortisol and DHEA Levels in the Korean Population: Age-Related Differences, Diurnal Rhythm, and Correlations with Serum Levels. Yonsei Med. J..

[67] Aizawa K., Iemitsu M., Maeda S., Otsuki T., Sato K., Ushida T., Mesaki N., Akimoto T. (2010). Acute exercise activates local bioactive androgen metabolism in skeletal muscle. Steroids.

[68] Heaney J.L., Carroll U., Phillips A.C. (2014). Physical activity, life events stress, cortisol, and DHEA: Preliminary findings that physical activity may buffer against the negative effects of stress. J. Aging Phys. Act..

[69] Flynn M.A., Weaver-Osterholtz D., Sharpe-Timms K., Allen S., Krause G. (1999). Dehydroepiandrosterone replacement in aging humans. J. Clin. Endocrinol. Metab..

[70] Jenkinson C., Petroczi A., Naughton D.P. (2013). Effects of dietary components on testosterone metabolism via UDP-glucuronosyltransferase. Front. Endocrinol..

